# Overexpression of EphB2 in the basolateral amygdala is crucial for inducing visceral pain sensitization in rats subjected to water avoidance stress

**DOI:** 10.1111/cns.14611

**Published:** 2024-02-14

**Authors:** Guang‐Bing Duan, Jun‐Wen Wang, Hui‐Hui Sun, Zhi‐Yu Dong, Yan Zhang, Zhen‐Xiang Wang, Ye Chen, Ying Chen, Ying Huang, Shu‐Chang Xu

**Affiliations:** ^1^ Department of Gastroenterology, Tongji Institute of Digestive Diseases, Tongji Hospital, School of Medicine Tongji University Shanghai China; ^2^ Key Laboratory of Spine and Spinal Cord Injury Repair and Regeneration (Ministry of Education), Department of Physiology and Pharmacology, Tongji Hospital, School of Medicine Tongji University Shanghai China

**Keywords:** basolateral amygdala, EphB2, irritable bowel syndrome, NMDA receptors, psychological stress, visceral hypersensitivity

## Abstract

**Aims:**

Basolateral amygdala (BLA), as a center for stress responses and emotional regulation, is involved in visceral hypersensitivity of irritable bowel syndrome (IBS) induced by stress. In the present study, we aimed to investigate the role of EphB2 receptor (EphB2) in BLA and explore the underlying mechanisms in this process.

**Methods:**

Visceral hypersensitivity was induced by water avoidance stress (WAS). Elevated plus maze test, forced swimming test, and sucrose preference test were applied to assess anxiety‐ and depression‐like behaviors. Ibotenic acid or lentivirus was used to inactivate BLA in either the induction or maintenance stage of visceral hypersensitivity. The expression of protein was determined by quantitative PCR, immunofluorescence, and western blot.

**Results:**

EphB2 expression was increased in BLA in WAS rats. Inactivation of BLA or downregulation of EphB2 in BLA failed to induce visceral hypersensitivity as well as anxiety‐like behaviors. However, during the maintenance stage of visceral pain, visceral hypersensitivity was only partially relieved but anxiety‐like behaviors were abolished by inactivation of BLA or downregulation of EphB2 in BLA. Chronic WAS increased the expression of EphB2, N‐methyl‐D‐aspartate receptors (NMDARs), and postsynaptic density protein (PSD95) in BLA. Downregulation of EphB2 in BLA reduced NMDARs and PSD95 expression in WAS rats. However, activation of NMDARs after the knockdown of EphB2 expression still triggered visceral hypersensitivity and anxiety‐like behaviors.

**Conclusions:**

Taken together, the results suggest that EphB2 in BLA plays an essential role in inducing visceral hypersensitivity. In the maintenance stage, the involvement of EphB2 is crucial but not sufficient. The increase in EphB2 induced by WAS may enhance synaptic plasticity in BLA through upregulating NMDARs, which results in IBS‐like symptoms. These findings may give insight into the treatment of IBS and related psychological distress.

## Graphical abstract

Overexpression of EphB2 in the basolateral amygdala may enhance synaptic plasticity through upregulating NMDARs, which results in visceral hypersensitivity and anxiety‐like behaviors in rats with psychological stress.

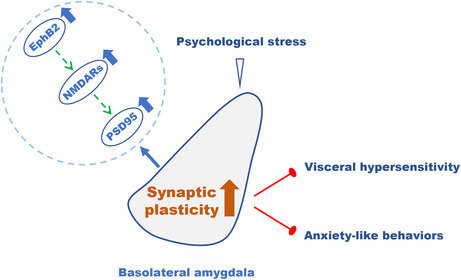

## 1 INTRODUCTION

Irritable bowel syndrome (IBS) is one of the most common diagnoses in gastroenterology, with the global prevalence at 3.8% according to Rome IV criteria.^1^, ^2^ It is a symptom‐based condition characterized by chronic abdominal pain or discomfort with altered stool form or frequency.^3^ Visceral hypersensitivity, defined by allodynia and hyperalgesia, is involved in pathophysiological processes leading to chronic visceral pain in IBS.^1^, ^4^ Growing evidence suggests that visceral hypersensitivity is a result of sensitization in peripheral and central nervous system.^5^ Psychiatric comorbidities including anxiety and depression are most prevalent in patients with IBS.^6^, ^7^ Supraspinal modulation of emotional state and psychological stress has significant effects on the perception of visceral pain.^8^, ^9^ Furthermore, psychological interventions such as cognitive behavioral therapies and gut‐directed hypnotherapy are efficacious for IBS in clinical practice.^10^, ^11^ These findings suggest a more widespread dysregulation of brain–gut interaction in the pathogenesis of IBS.

Altered activation of brain regions responsible for cognitive processing and emotional responses to visceral stimuli has been found in patients with IBS.^5^, ^12^, ^13^, ^14^ Among them, basolateral amygdala (BLA), an almond‐shaped neural substrate in medial temporal lobe, plays a critical role in stress responses and emotional regulation.^15^, ^16^, ^17^ A growing body of evidence suggests that the amygdala plays an important role in determining the negative affective qualities of acute and chronic pain.^18^, ^19^, ^20^ Moreover, it is reported that enhancement of synaptic plasticity in BLA facilitates the development of pain chronicity and comorbid mental symptoms in neuropathic pain.^21^, ^22^ However, the roles of BLA in visceral pain and the underlying mechanisms of IBS are still poorly understood.

As the largest family of receptor tyrosine kinases (RTKs), Eph receptors and their membrane‐bound ligands Ephrin are emerging key players in the development of neuronal networks.^23^ In mature brain synapses, EphB2 receptor (EphB2) as an important Eph receptor, is highly expressed in large dendritic shafts and spines in the frontal cortex, hippocampus, and amygdala.^24^ EphB2 has been found to modulate synaptogenesis and synaptic plasticity, especially in excitatory glutamatergic synapses.^25^, ^26^ Moreover, EphB2 also participates in various pathological processes. For instance, it has been reported that activation of EphB2 in BLA promotes stress vulnerability in mice and induces depression‐like behaviors.^27^ Overexpression of EphB2 in the hippocampus ameliorates impaired N‐methyl‐D‐aspartate receptors (NMDARs) trafficking and rescues cognitive dysfunction in a rodent model of Alzheimer's disease.^28^ In spinal cord neurons, EphB2 activation contributes to nociceptive dorsal root ganglion (DRG) neurons' hyperexcitability through interaction with NMDARs in neuropathic pain.^29^ In enteric nervous system, EphB2 activation facilitates synaptic potentiation, leading to visceral hypersensitivity in IBS.^30^, ^31^ However, whether EphB2 in BLA is involved in visceral hypersensitivity remains to be elucidated.

In the present study, we aimed to explore the role of BLA in the induction and maintenance stage of stress‐induced visceral hypersensitivity, and then investigate the underlying mechanisms of EphB2 that may be involved in these processes.

## 2 METHODS

### 2.1 Animals

The male Wistar rats (180–250 g) were supplied by SPF (SPF Biotechnology Co., Ltd, Soochow, China). Rats were housed in groups of four to six per cage under a constant temperature (24 ± 1°C) and maintained on a 12‐h light/dark cycle. Rats were used for experiments after 7 days of acclimatization.

### 2.2 Visceral hypersensitivity rat model

The visceral hypersensitivity model was established by chronic water avoidance stress (WAS) as previously described.^32^ Briefly, a platform was placed in the center of a tank, and rats in WAS group were placed on the platform for an hour each day for 10 days with the water surface in the tank 1 cm below the platform top. Rats in Sham‐WAS group were placed on the platform but without water. Rats in normal control (NC) group were left undisturbed.

### 2.3 Visceromotor response (VMR) assessment

The VMR to colorectal distension (CRD) was recorded by the abdominal muscle electromyogram (EMG) as previously described.^33^ In brief, after anesthetized with intraperitoneal injection of pentobarbital sodium (50 mg/kg), a pair of silver wires were implanted into the left external abdominal oblique muscle. Rats were individually housed for 3 days following surgery. Then, a certain pressure (40 or 60 mmHg) CRD was maintained in the colorectal cavity by gas injection. Three cycles of CRD (20‐s duration; 5‐min interstimulus interval for recovery) were conducted in each rat. The area under the curve of prestimulation and stimulation period were compared.

### 2.4 Behavioral tests

#### 2.4.1 Elevated plus maze (EPM)

Anxiety level of rats was tested by EPM. The EPM apparatus consists of two open arms (50 cm length × 10 cm width) and two closed arms (50 cm length × 10 cm width × 40 cm height), raised 70 cm off the ground. The percentage of duration in the open arms and the percentage of frequency of entries into the open arms were analyzed. The anxiety index was also calculated to evaluate anxiety levels. Anxiety index = 1 − [(time spent in open arms/total time on the maze + number of entries to the open arms/total exploration on the maze)/2]. The apparatus was swabbed with 75% ethanol after each test.

#### 2.4.2 Sucrose preference test (SPT)

SPT is usually applied to evaluate anhedonia symptoms in animals, and a decrease in the sucrose consumption ratio is indicative of depression‐like behaviors.^34^ Rats were trained to drink two bottles of 1% sucrose solution for the first 24 h. One bottle of 1% sucrose was changed with pure water on the following day. To prevent the possible effect of side preference in drinking behavior, bottle position was changed every 8 h. Subsequently, rats were deprived of food and water for 24 h and then were presented with food and two preweighed bottles of drinking solution (one with 1% sucrose solution and the other with pure water). Three hours later, bottles were reweighed and the consumptions of sucrose solution and water were calculated. Sucrose preference (%) = sucrose solution consumption/(sucrose solution consumption + water consumption) × 100.

#### 2.4.3 Forced swimming test (FST)

FST was performed to assess behavioral despair in animals, indicating a kind of depression‐like behavior. In brief, rats were placed individually into a water‐filled transparent cylinder (30 cm in diameter, 80 cm in water height, and water temperature: 24 ± 1°C) and their movement was monitored for 6 min. Immobility time was recorded in the last 4 min. Immobility was determined as a rat remained floating in the water making only movements necessary to keep its head above the water.

### 2.5 Stereotaxic surgery and intra‐BLA microinjection

After anesthetized, rats were fixed in a stereotaxic instrument (David Kopf Instruments, USA). A 33‐gauge infusion needle (Hamilton, USA) was targeted to bilateral BLA at the following coordinates: anteroposterior, −2.8 mm from bregma; mediolateral, ±4.8 mm; and dorsoventral, 8 mm ventrally from the skull surface. Drug was infused with 0.08 μL/min using a syringe pump (Harvard Apparatus, USA). Before extraction, the needle was raised by 0.1 mm and held for 5 min to allow diffusion.

Inactivation of BLA was conducted using ibotenic acid (IBO) (HY‐N2311, MedChemExpress, USA). IBO solution (10 μg/μL) was prepared freshly in phosphate‐buffered saline (PBS, pH 7.4). Sham‐lesion of BLA was performed similarly but with PBS infusion. IBO solution or PBS was microinjected with 0.3 μL on each side. Rats were cared to recover for a week before experiments. Coronal brain sections (25 μm thick) were stained with Nissl (C0117, Beyotime Biotechnology, China) for histology confirmation of lesion location and spread area in BLA by microscopic inspection (Olympus BX53, Japan).

Lentivirus pCLenti‐U6‐shRNA (EphB2)‐CMV‐EGFP (shEphB2, 5 × 10^8^ transducing units [TU]/mL) was used to downregulate the expression of EphB2. Instead, Sham lentivirus used in the control group was pCLenti‐U6‐shRNA (scramble)‐CMV‐EGFP (Sham‐virus, 5 × 10^8^ TU/mL). Both of them were purchased from Obio Technology (Shanghai, China). Lentivirus was microinjected with 0.8 μL in each side of BLA. Rats were cared to recover for a week before experiments. Brain sections of BLA were collected according to the stereotaxic atlas^35^ to verify virus expression.

Cannulation was performed for the experiment of NMDAR activation as previously described.^32^ Briefly, two 9.2 mm stainless steel cannulas (24‐gauge, RWD Life Science Co., Ltd. China) were implanted above BLA. The shEphB2 virus (0.8 μL/side) was infused through a 28‐gauge inner cannula which was connected to a syringe pump. After the virus microinjection and WAS, rats were then microinjected with the selective NMDARs agonist NMDA (0.3 μL/side,1 μg/μL dissolved in PBS; S7072, Selleck, USA) or PBS (0.3 μL/side) through the cannulas. Behavioral tests and VMR assessment were performed 20 min after the NMDA or PBS infusion.

### 2.6 Quantitative real‐time PCR (qRT‐PCR)

As described previously, total RNA was collected and reverse transcribed into cDNA.^33^ Expression levels of target genes were analyzed by qPCR Master Mix (G3326, Wuhan Servicebio Technology, China). The quantification of target gene was standardized by GAPDH and the expression levels of the target gene were analyzed by △△Ct method. Primers (Sangon, China) used are shown in Table 1.

**TABLE 1:** Primer sequences used for qRT‐PCR analysis.

Gene name	Sequence (5′ → 3′)	
	Forward	Reserve
GAPDH	ACGGCAAGTTCAACGGCACAG	CGACATACTCAGCACCAGCATCAC
EphA4	GGAAGGAGGGTGGGAGGAAGTAAG	AGTCAGTTCGCAGCCAGTTGTTC
EphA5	AAGCAAACTTCGGCGGTCTCTG	GCGTGGTTCAGTCTAAGGTTCAGG
EphA6	GTTACCGACTTCCTGCTCCAATGG	GCCTGTGGTTTCTCTCCTTCTGC
EphA7	TTGAAGCAACAGCGGTGTCCAG	ACCACAGTGCCTTCTTCCAATGATG
EphB1	AGAAAGGAAGGAGGTGGAGGGAAG	AGGGAGGAGTACGAATGGAGGAAAG
EphB2	ATGGGCGTTACAGTGGCAAGATG	CACGGCGATGACGAAGACTACG
EfnA3	GTCTGAGGATGAAGGTGTTCGTCTG	GCTGGTTCCACTGATGCTCTTCTC
EfnB2	TGGTTGATAAAGAGCAAGCCGACAG	CTTGGTCTGGTCTGGCACAGTTG
EfnB3	TGAGAAGGTGAGTGGTGACTACGG	TGGGCGACTGGATTGAGGAGAG

### 2.7 Western blot

Protein (20 μg/lane) collected from bilateral BLA was subjected to SDS‐PAGE electrophoresis and subsequently transferred onto PVDF membranes, and incubated at 4°C overnight with following primary antibody: EphB2 (1:1000, ab252935, Abcam), NR2B (1:1000, ab65783, Abcam), NR2A (1:1000, ab124913, Abcam), NR1 (1:1000, ab109182, Abcam), postsynaptic density protein (PSD95, 1:1000, 3450S, Cell Signaling Technology), or β‐Tubulin (1:1000, ARG62347, Arigo Biolaboratories Co.). Membranes were washed and incubated with the secondary antibody (1:5000, ARG65350 or ARG65351, Arigo Biolaboratories Co.) with a horseradish peroxidase label for an hour at room temperature. The bands were visualized by a chemiluminescence imaging system (GE Healthcare, USA) with enhanced chemiluminescence reagents (Millipore, USA). Analysis of the gel image was performed using ImageJ software. β‐Tubulin was used as a reference protein.

### 2.8 Immunofluorescence staining

Brain slices of (25 μm thickness) were rinsed with PBS 5 min for three times and then incubated for penetrating with permeable buffer for 15 min and blocked with blocking buffer (P0260, Beyotime Biotechnology, China) for 15 min at room temperature. Immunofluorescence staining was conducted using the following primary antibodies: a mixture of NeuN (1:50, ab177487, Abcam) and c‐fos (1:50, GB11069, Wuhan Servicebio Technology, China) for immunofluorescence double labeling; or EphB2 (1:50, ab252935, Abcam). Primary antibody incubation was performed at 4°C overnight. The slices were rinsed and then incubated with Alexa Fluor secondary antibodies specific for the primary antibody host (Beyotime, China) for 2 h.

### 2.9 Statistical analysis

The statistical analysis was conducted with GraphPad Prism (version 9.4.1, GraphPad Software, USA). Data were presented as mean ± standard error of mean (SEM). Shapiro–Wilk test was used for normality test (*α* = 0.05). Unpaired Student's *t* test was used to analyze data of qRT‐PCR, western blot, and immunofluorescence. One‐way ANOVA followed by the Tukey's post hoc test or Dunnett's post hoc test was used to analyze data of VMR assessment or behavioral tests. Mann–Whitney test or Kruskal–Wallis test was used to analyze the non‐normal distribution data. *p* < 0.05 was considered to be statistically significant.

## 3 RESULTS

### 3.1 BLA was activated in rats with visceral hypersensitivity induced by WAS

**FIGURE 1:**
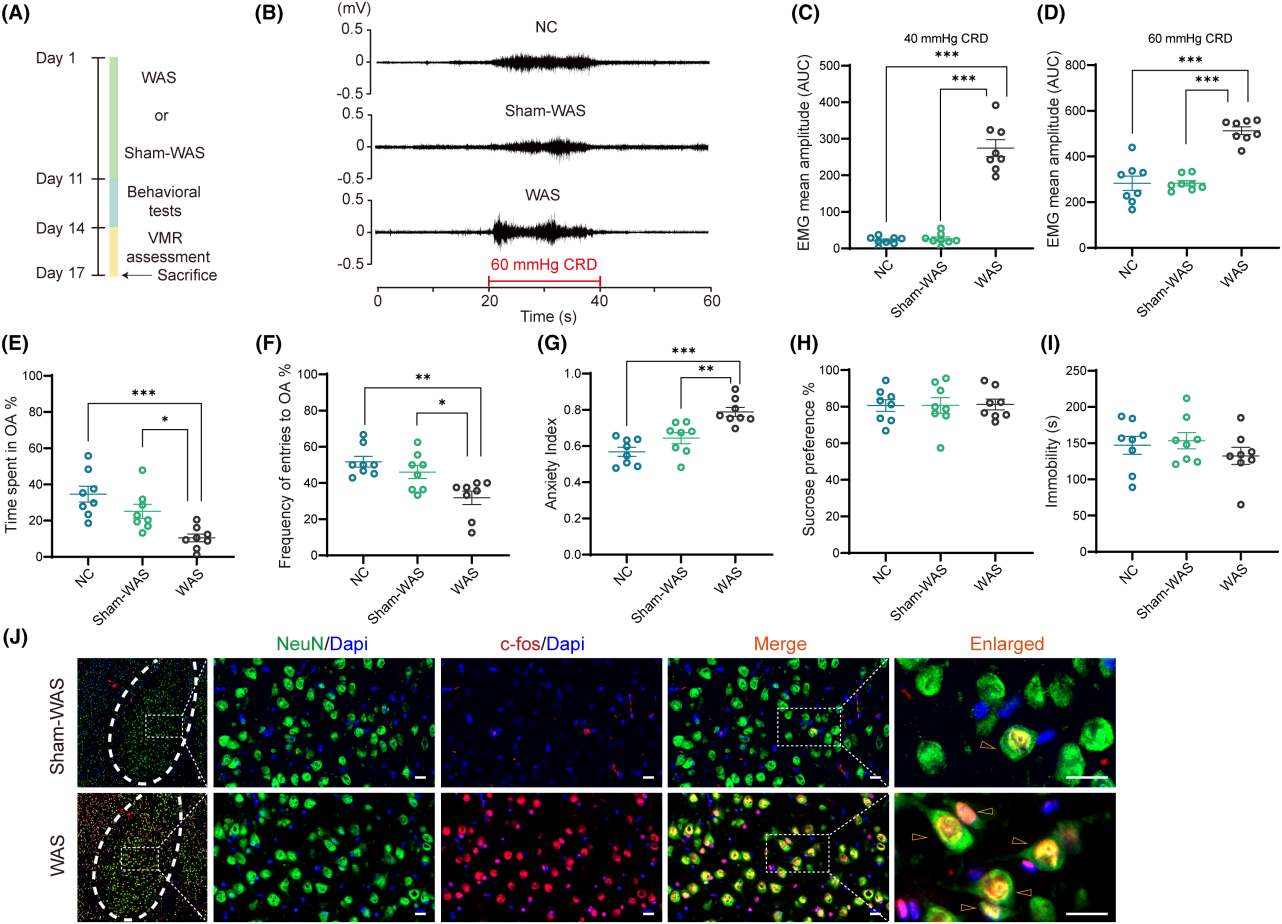
Basolateral amygdala (BLA) was activated in rats with visceral hypersensitivity induced by water avoidance stress (WAS). (A) A schematic representation of the experimental protocol. (B) Representative electromyogram (EMG) recordings of visceromotor response (VMR) amplitudes to 60 mmHg colorectal distension (CRD) in rats of normal control (NC), Sham‐WAS, and WAS. VMR amplitudes to both (C) 40 mmHg and (D) 60 mmHg CRD were increased in rats of WAS compared with that in NC and Sham‐WAS groups. No significant difference was detected in VMR amplitudes between rats in NC and Sham‐WAS groups. In elevated plus maze (EPM) test, rats in WAS group (E) spent less time in open arms (OA) and (F) made less entries into OA compared with other groups. (G) Rats in WAS had a higher anxiety index than that in other two groups. There was no difference in anxiety‐like behaviors between rats in NC and Sham‐WAS groups. No significant difference was detected (H) in the sucrose preference in sucrose preference test (SPT) or (I) in the immobility time in forced swimming test (FST) among these three groups. *n* = 8 for each group. All data are given as mean ± SEM. *p < 0.05, **p < 0.01, ***p < 0.001, one‐way ANOVA followed by Tukey's or Dunnett's post hoc test. (J) Immunofluorescent staining showed that the expression of the immediate‐early gene c‐fos in BLA neurons was increased in WAS rats compared with Sham‐WAS group. Scale bar: 20 μm.

VMR assessment was conducted to evaluate visceral sensitivity in Wistar rats. The detailed experimental design is shown in Figure 1A. A representative EMG recording of VMR amplitudes at 60 mmHg CRD is shown in Figure 1B. VMR amplitudes to 40 or 60 mmHg CRD significantly increased in rats of WAS group compared with that in NC and Sham‐WAS groups (Figure 1C,D; *n* = 8 for each group, one‐way ANOVA with Welch's correction, followed by Dunnett's post hoc test; C, *p* < 0.0001; D, *p* < 0.0001). However, between NC and Sham‐WAS rats, no significant difference in VMR amplitudes was detected (Figure 1C,D; *n* = 8 for each group).

Meanwhile, as WAS is a psychiatric stress, the EPM was applied to test anxiety levels of the rats. Compared with rats in NC and Sham‐WAS groups, WAS rats spent less time and made significantly fewer entries into the open arms, and had a higher anxiety index (Figure 1E–G; *n* = 8 for each group, one‐way ANOVA followed by Tukey's post hoc test; E, *F*_(2, 21)_ = 11.30, *p* = 0.0005; F, *F*_(2, 21)_ = 8.757, *p* = 0.0017; G, *F*_(2, 21)_ = 17.00, *p* < 0.0001). Between NC and Sham‐WAS rats, no significant difference in anxiety‐like behaviors was detected (Figure 1E–G; *n* = 8 for each group). In addition, as depression is a common comorbid condition with anxiety, SPT and FST were used to test whether WAS could also induce depression‐like behaviors. However, the difference in the percentage of sucrose consumption in SPT or the immobility time in FST was not detected among these three groups (Figure 1H,I; *n* = 8 for each group, one‐way ANOVA followed by Tukey's post hoc test; H, *F*_(2, 21)_ = 0.0081, *p* = 0.9919; I, *F*_(2, 21)_ = 0.8340, *p* = 0.4482). The results suggest that chronic WAS induces visceral hypersensitivity and anxiety‐like behaviors.

Because no significant difference in VMR amplitudes or anxiety‐like behaviors was detected between rats in NC and Sham‐WAS groups, Sham‐WAS group was set as a control group in the following experiments.

Since BLA is critical for regulation of anxiety and pain, whether WAS activated the neurons in BLA was subsequently investigated. As c‐fos is the neuronal activation marker, its expression in BLA neurons was measured. It was found that c‐fos expression in BLA increased after chronic WAS compared with that in Sham‐WAS group (control) (Figure 1J). These results suggest that BLA may participate in the pathological process of stress‐related visceral hypersensitivity.

### 3.2 BLA lesion prior to WAS failed to induce visceral hypersensitivity and anxiety‐like behaviors

**FIGURE 2:**
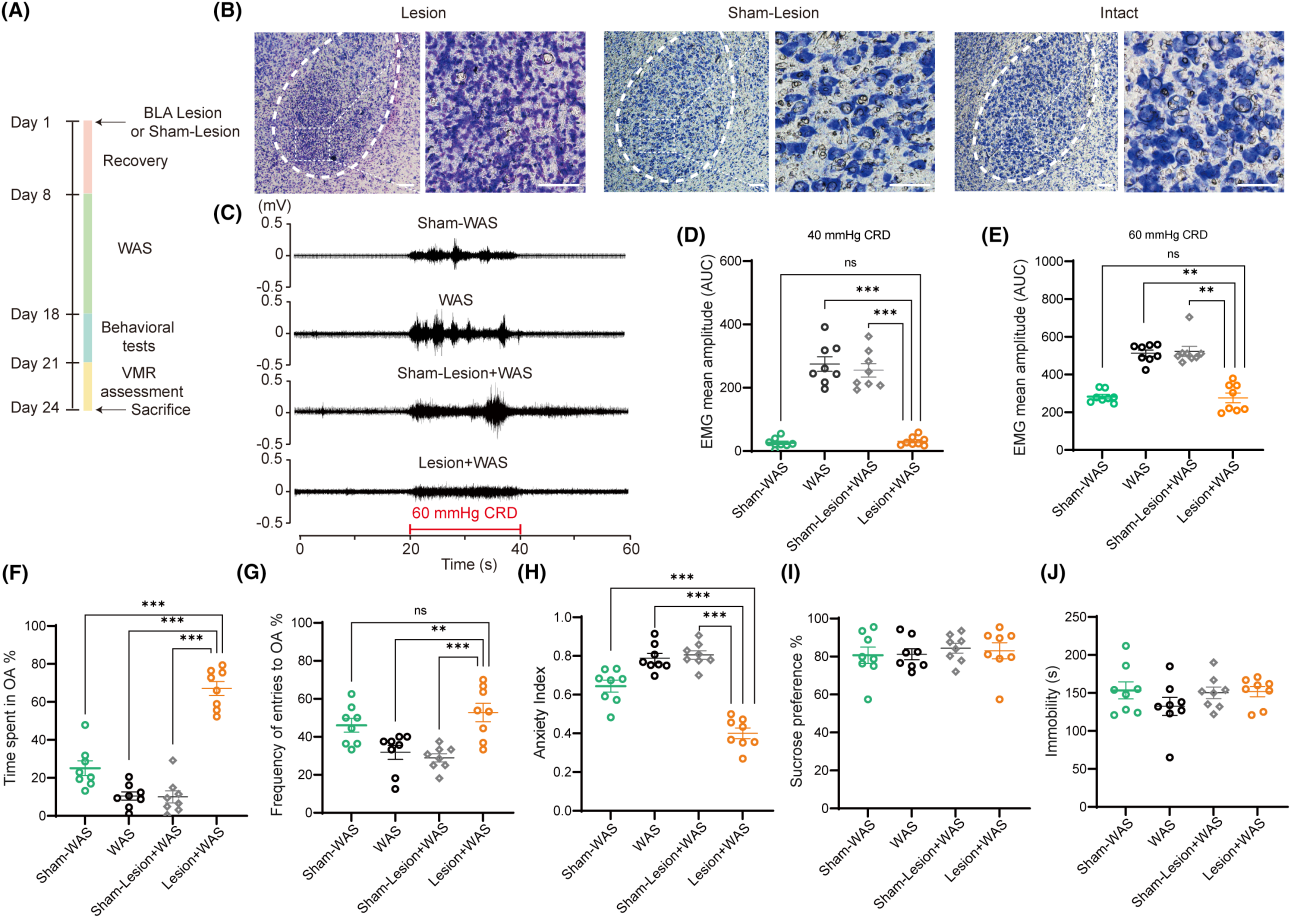
BLA lesion prior to WAS failed to induce visceral hypersensitivity and anxiety‐like behaviors in WAS rats. (A) A schematic of the protocol for the experiments. (B) The representative photomicrographs of BLA lesion (left, IBO‐infused), Sham‐Lesion (middle, PBS infused), and intact (right, without infusion). Dispersion and dissolution of Nissl substance were observed in BLA infused with IBO compared with that in other two groups. (C) The representative EMG recordings of VMR amplitudes to 60 mmHg CRD. Rats in Lesion + WAS group showed lower VMR amplitudes to (D) 40 mmHg and (E) 60 mmHg CRD compared with rats in WAS and Sham‐Lesion + WAS groups. There was no difference in VMR amplitudes to 40 or 60 mmHg CRD between rats in Lesion + WAS group and Sham‐WAS groups. In EPM test, rats in Lesion + WAS group (F) spent more time in OA and (G) made more entries into OA compared with that in WAS and Sham‐Lesion + WAS groups. (H) Rats in Lesion + WAS group had a lower anxiety index than that in other three groups. There was no significant difference (I) in the sucrose preference in SPT or (J) in the immobility time in FST among these four groups. *n* = 8 for each group. All data are given as mean ± SEM. **p < 0.01, ***p < 0.001, one‐way ANOVA followed by Tukey's post hoc test or Kruskal–Wallis test. Scale bar: 200 μm.

To investigate if BLA is crucial to visceral hypersensitivity induced by WAS, bilateral BLA were inactivated by IBO microinjection 7 days before WAS. The detailed experimental design is shown in Figure 2A. BLA with IBO infusion extended from bregma −1.72 to −2.92 mm according to *Paxinos* and *Waston*.^35^ Data from five lesioned animals were excluded for analysis due to the lesion outside BLA or too small lesion size. The representative photomicrographs of BLA lesion (left, IBO infused), Sham‐lesion (middle, PBS infused) and intact (right, without infusion) are shown in Figure 2B. Dispersion and dissolution of Nissl substance were observed in BLA infused with IBO compared with that in other two groups. A representative EMG recording of VMR amplitudes at 60 mmHg CRD is shown in Figure 2C. Compared with rats in WAS and Sham‐Lesion + WAS groups, rats in bilateral BLA lesion before WAS (Lesion + WAS group) showed lower VMR amplitudes to 40 or 60 mmHg CRD (Figure 2D,E; *n* = 8 for each group, one‐way ANOVA followed by Tukey's post hoc test or Kruskal–Wallis test; D, *F*_(3, 28)_ = 71.90, *p* < 0.0001; E, *p* < 0.01). Between WAS and Sham‐Lesion + WAS rats, no significant difference in VMR amplitudes was detected (Figure 2D,E; *n* = 8 for each group). Furthermore, rats in Lesion + WAS group had similar VMR amplitudes to 40 or 60 mmHg CRD compared with that in Sham‐WAS group (control) (Figure 2D,E; *n* = 8 for each group). The results suggest that bilateral BLA lesion prior to WAS fails to induce visceral hypersensitivity.

In the EPM test, rats in Lesion + WAS group spent more time, made more entries into the open arms, and had a lower anxiety index compared with that in WAS and Sham‐Lesion + WAS groups (Figure 2F–H; *n* = 8 for each group, one‐way ANOVA followed by Tukey's post hoc test; F, *F*_(3, 28)_ = 66.49, *p* < 0.0001; G, *F*_(3, 28)_ = 9.374, *p* = 0.0002; H, *F*_(3, 28)_ = 50.27, *p* < 0.0001). Furthermore, compared with rats in Sham‐WAS group (control), rats in Lesion + WAS group spent more time, made similar entries into the open arms, and had an even lower anxiety index (Figure 2F–H; *n* = 8 for each group, one‐way ANOVA followed by Tukey's post hoc test; F, *F*_(3, 28)_ = 66.49, *p* < 0.0001; G, *F*_(3, 28)_ = 9.374, *p* = 0.0002; H, *F*_(3, 28)_ = 50.27, *p* < 0.0001). The results of EPM suggest that lesion of BLA before WAS fails to induce anxiety‐like behaviors. There was no significant difference in the percentage of sucrose consumption in SPT or in the immobility time in FST among these groups (Figure 2I,J; *n* = 8 for each group, one‐way ANOVA followed by Tukey's post hoc test; I, *F*_(3, 28)_ = 0.2243, *p* = 0.8787; J, *F*_(3, 28)_ = 1.041, *p* = 0.3898).

Therefore, these data suggest that BLA is necessary to induce visceral hypersensitivity and anxiety‐like behaviors in WAS rats.

### 3.3 BLA lesion after WAS alleviated visceral hypersensitivity and anxiety‐like behaviors

**FIGURE 3:**
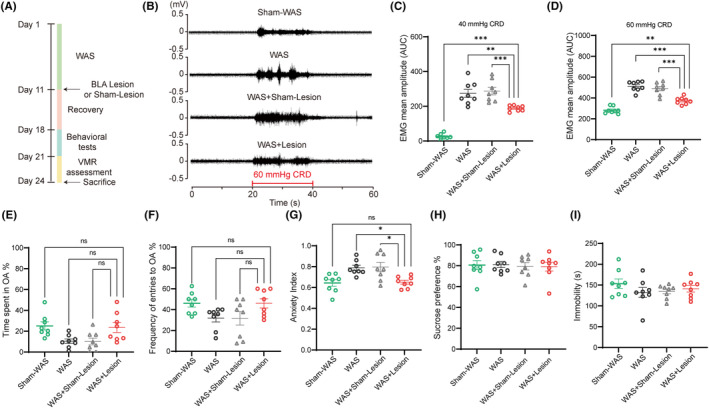
BLA lesion after WAS only partially alleviated visceral hypersensitivity but abolished anxiety‐like behaviors in WAS rats. (A) The detailed experimental protocol of this section. (B) The representative EMG recordings of VMR amplitudes to 60 mmHg CRD. Rats in WAS + Lesion group showed lower VMR amplitudes to (C) 40 mmHg and (D) 60 mmHg CRD. However, rats in WAS + Lesion group still had higher VMR amplitudes compared with that in Sham‐WAS group. In EPM test, no significant difference was detected (E) in the percentage of time spent in OA or (F) in the percentage of frequency of entries to OA in WAS + Lesion group compared with other three groups. However, (G) rats in WAS + Lesion group still had a lower anxiety index than that in WAS and WAS + Sham‐Lesion groups, and had a similar anxiety index with Sham‐WAS group. There was no significant difference (H) in the sucrose preference in SPT or (I) in the immobility time in FST among these four groups. *n* = 8 for each group. All data are given as mean ± SEM. *p < 0.05, **p < 0.01, ***p < 0.001, one‐way ANOVA followed by Tukey's post hoc test.

Lesion or Sham‐Lesion of bilateral BLA was applied after chronic WAS to further investigate the role of BLA in the maintenance of visceral hypersensitivity induced by WAS. The detailed experimental protocol of this section is shown in Figure 3A. The representative EMG recordings of VMR amplitudes to 60 mmHg CRD are shown in Figure 3B. Rats with bilateral BLA lesion after WAS (WAS + Lesion group) showed lower VMR amplitudes to 40 and 60 mmHg CRD compared with that in WAS and WAS + Sham‐Lesion groups (Figure 3C,D; *n* = 8 for each group, one‐way ANOVA followed by Tukey's post hoc test; C, *F*_(3, 28)_ = 55.20, *p* < 0.0001; D, *F*_(3, 28)_ = 46.36, *p* < 0.0001). Between WAS and WAS + Sham‐Lesion rats, no significant difference in VMR amplitudes was detected (Figure 3C,D; *n* = 8 for each group). However, WAS + Lesion rats still showed higher VMR amplitudes to 40 or 60 mmHg compared with that in Sham‐WAS group (control) (Figure 3C,D; *n* = 8 for each group, one‐way ANOVA followed by Tukey's post hoc test; C, *F*_(3, 28)_ = 55.20, *p* < 0.0001; D, *F*_(3, 28)_ = 46.36, *p* < 0.0001). The results suggest that bilateral BLA lesion after WAS only partially alleviates visceral hypersensitivity in WAS rats.

In the EPM test, no significant difference was detected in time spent or entries made into the open arms in WAS + Lesion rats compared with other three groups (Figure 3E,F; *n* = 8 for each group). However, WAS + Lesion rats still had a lower anxiety index compared with rats in WAS and WAS + Sham‐Lesion groups, and had a similar anxiety index compared with Sham‐WAS rats (control), suggesting that lesion of BLA after WAS abolishes anxiety‐like behaviors (Figure 3G; *n* = 8 for each group, one‐way ANOVA followed by Tukey's post hoc test; *F*_(3, 28)_ = 7.119, *p* = 0.0011). Consistently, none of these four groups of rats showed significant difference in the percentage of sucrose consumption in SPT or in the immobility time in FST (Figure 3H,I; *n* = 8 for each group, one‐way ANOVA followed by Tukey's post hoc test; H, *F*_(3, 28)_ = 0.0615, *p* = 0.9796; I, *F*_(3, 28)_ = 0.9760, *p* = 0.4180).

Taken together, these results suggest that BLA also participates in the maintenance of visceral hypersensitivity and anxiety‐like behaviors induced by WAS.

### 3.4 The expression of EphB2 and NMDARs in BLA was increased in WAS rats

**FIGURE 4:**
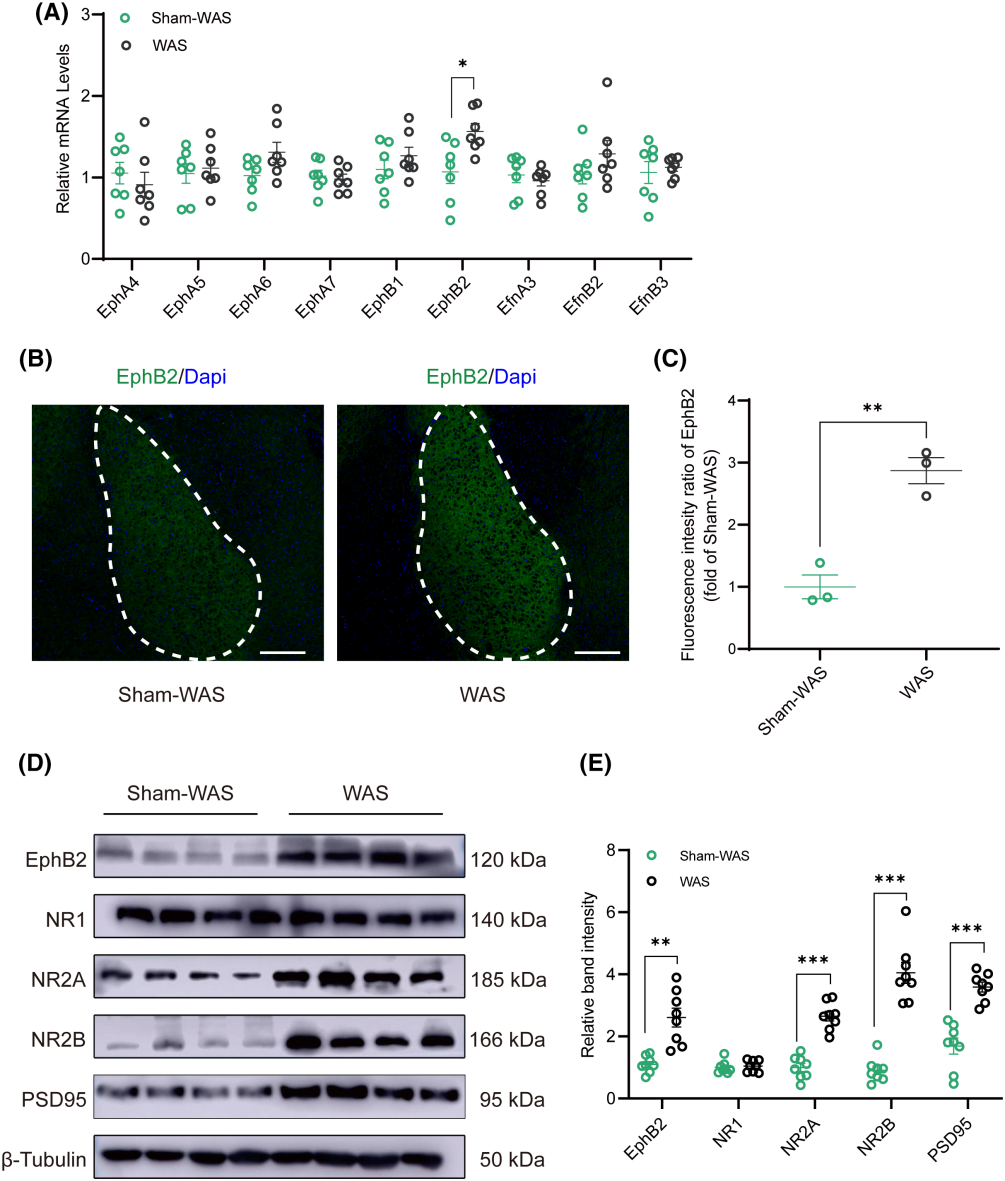
WAS increased the expression of EphB2 and NMDARs in BLA. (A) The mRNA expression of EphB2 in BLA was significantly higher in WAS rats than that in Sham‐WAS rats. No difference was detected in the mRNA expression of other receptors or ligands of Eph family. *n* = 7 for each group. All data are given as mean ± SEM. *p < 0.05, two‐tailed unpaired Student's *t*‐test. (B) Representative fluorescence image showed the expression of EphB2 (green) in BLA in rats of Sham‐WAS (left) and WAS (right). Scale bar: 100 μm. (C) Quantification of the mean gray value of EphB2 in BLA showed higher expression in WAS rats compared with that in Sham‐WAS group. *n* = 3 rats for each group, six sections for each rat. All data are given as mean ± SEM. **p < 0.01, two‐tailed unpaired Student's *t*‐test. (D) Representative immunoblots showed the expression of EphB2, NR1, NR2A, NR2B, PSD95, and β‐Tubulin in Sham‐WAS and WAS groups. (E) The protein expression of EphB2, NR2A, NR2B, and PSD95 in BLA was increased in rats of WAS group than that of Sham‐WAS group. *n* = 8 for each group. All data are given as mean ± SEM. **p < 0.01, ***p < 0.001, two‐tailed unpaired Student's *t*‐test.

Given that Eph‐Ephrin system controls many cellular processes and is involved in the modulation of pain, qRT‐PCR was conducted to identify whether Eph receptors and their ligands (EfnA3, EfnB2, and EfnB3) in BLA were influenced by chronic WAS. The results showed that only EphB2 was significantly upregulated in WAS rats compared with Sham‐WAS rats (Figure 4A; *n* = 7 for each group, unpaired Student's *t* test; EphB2, *t*_(12)_ = 2.871, *p* = 0.0141). Subsequently, in situ immunofluorescent analysis showed that the mean intensity of EphB2 protein in BLA was stronger in WAS rats than that in Sham‐WAS rats (Figure 4B,C; *n* = 3 rats for each group, 6 sections for each rat, unpaired Student's *t* test; C, *t*_(4)_ = 6.576, *p* = 0.0028). It has been reported that EphB2 enhanced long‐term potentiation (LTP) through interacting with NMDARs. Western blot was conducted to verify the protein expression of EphB2 and NMDARs. The results showed that expression of EphB2, NR2A, and NR2B was significantly increased in WAS rats compared with Sham‐WAS rats (Figure 4D,E; *n* = 8 for each group, unpaired Student's *t* test; E, EphB2, Welch's correct, *t*_(8.283)_ = 4.737, *p* = 0.0013; NR2A, *t*_(14)_ = 8.112, *p* < 0.0001; NR2B, Welch's correct, *t*_(9.253)_ = 8.634, *p* < 0.0001), while the expression of NR1 was similar between Sham‐WAS and WAS rats (Figure 4D,E; *n* = 8 for each group, unpaired Student's *t* test; *t*_(14)_ = 0.4366, *p* = 0.6691). Furthermore, as one of the markers of synaptic plasticity, PSD95 expression significantly increased in WAS rats (Figure 4D,E; *n* = 8 for each group, unpaired Student's *t* test; *t*_(14)_ = 6.239, *p* < 0.0001). These data suggest that chronic WAS increases the expression of EphB2 as well as NMDARs in BLA and enhances synaptic plasticity.

### 3.5 EphB2 knockdown in BLA before WAS failed to induce visceral hypersensitivity and anxiety‐like behaviors

**FIGURE 5:**
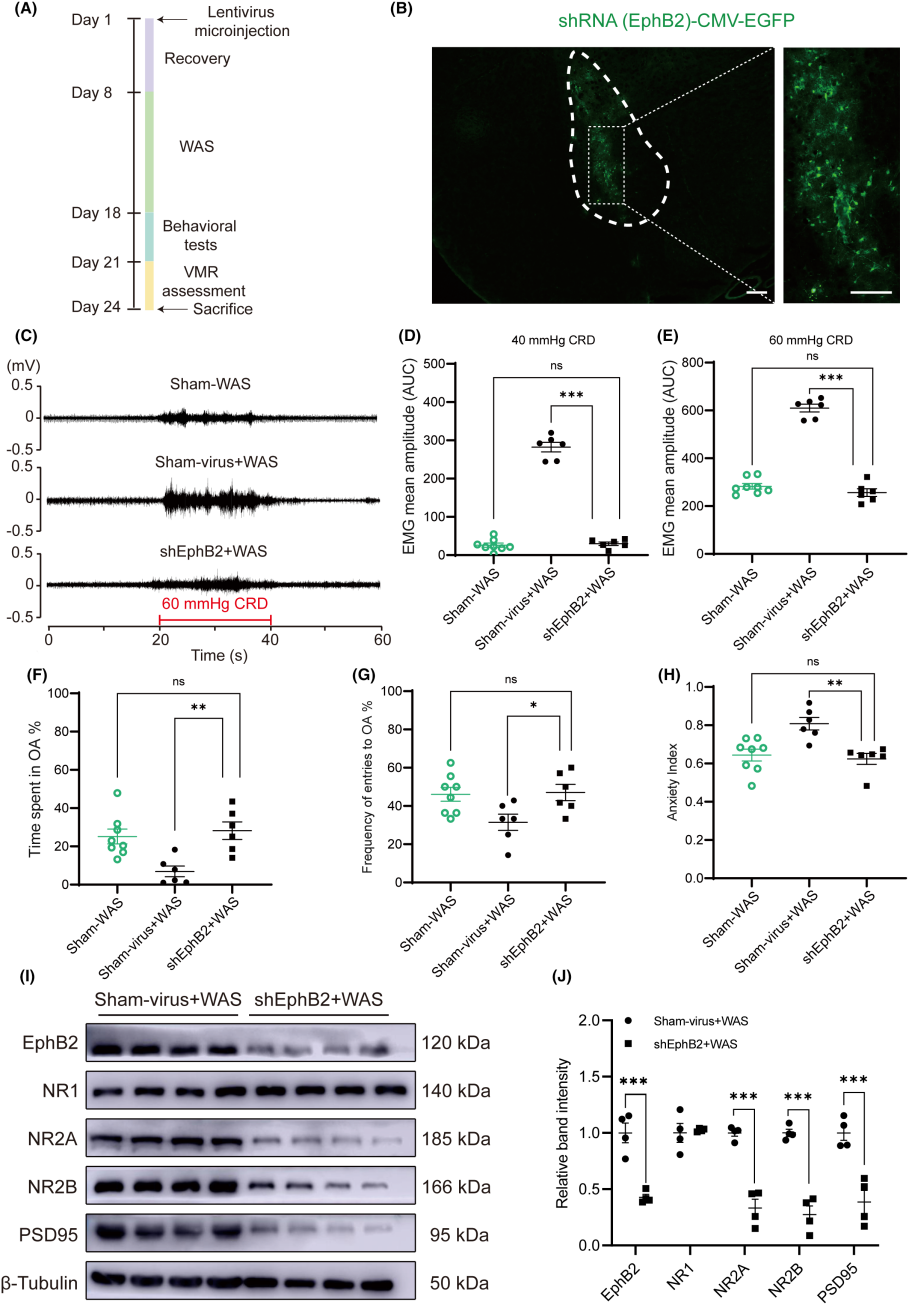
EphB2 knockdown in BLA before WAS failed to induce visceral hypersensitivity and anxiety‐like behaviors. (A) The schematic representation of the experimental protocol. (B) A representative fluorescence image confirmed the expression of lentivirus in shEphB2‐infected cells (green) in BLA. Scale bar, 200 μm. (C) The representative EMG recordings of VMR amplitudes to 60 mmHg CRD. Rats of shEphB2 + WAS had lower VMR amplitudes to (D) 40 mmHg and (E) 60 mmHg CRD compared with that in Sham‐virus + WAS groups. Moreover, there was no difference in VMR amplitudes to 40 or 60 mmHg CRD between rats in Sham‐WAS and shEphB2 + WAS. In EPM test, rats in shEphB2 + WAS group (F) spent more time in OA and (G) made more entries into OA than that in Sham‐virus + WAS group. (H) Rats in shEphB2 + WAS group had a lower anxiety index than that in Sham‐virus + WAS group. *n* = 6 for each group. No significant difference was detected in anxiety‐like behaviors between rats in Sham‐WAS and shEphB2 + WAS. *n* = 8 for Sham‐WAS group, *n* = 6 for another groups. All data are given as mean ± SEM. *p < 0.05, **p < 0.01, ***p < 0.001, one‐way ANOVA followed by Tukey's post hoc test or Kruskal–Wallis test. (I) Representative immunoblots showed the expression of EphB2, NR1, NR2A, NR2B, PSD95, and β‐tubulin. (J) The protein expression of EphB2, NR2A, NR2B, and PSD95 in BLA was decreased in shEphB2 + WAS group compared with Sham‐virus + WAS. *n* = 4 for each group. All data are given as mean ± SEM. *p < 0.05, **p < 0.01, and ***p < 0.001, two‐tailed unpaired Student's *t*‐test.

Lentiviral intervention was conducted to knock down the EphB2 protein expression in WAS rats to further figure out the relationship between EphB2 in BLA and the induction of visceral hypersensitivity. The experimental protocol of this part is shown in Figure 5A. A representative fluorescence image confirming the expression of lentivirus with green fluorescent protein (GFP) in shEphB2‐infected cells in BLA is shown in Figure 5B. The virus spanned BLA from bregma −1.92 to −2.92 mm according to atlas.^35^ Compared with rats injected with Sham lentivirus in bilateral BLA before WAS (Sham‐virus + WAS group), rats microinjected with shEphB2 lentivirus before WAS (shEphB2 + WAS group) showed significantly lower visceral sensitivity (Figure 5C–E; C, VMR amplitudes to 60 mmHg CRD; *n* = 6 for shEphB2 + WAS or Sham‐virus + WAS group, one‐way ANOVA followed by Tukey's post hoc test; D, *F*_(2, 17)_ = 330.1, *p* < 0.0001; E, *F*_(2, 17)_ = 177.0, *p* < 0.0001). Furthermore, there was no difference in VMR amplitudes to 40 or 60 mmHg CRD between rats in shEphB2 + WAS and Sham‐WAS groups (control) (Figure 5D,E; *n* = 8 for Sham‐WAS group, *n* = 6 for shEphB2 + WAS group). The results suggest that knocking down the protein of EphB2 in bilateral BLA before WAS fails to induce visceral hypersensitivity in WAS rats. In the EPM test, compared with Sham‐virus + WAS group, rats in shEphB2 + WAS group spent more time in the open arms, made more entries into the open arms, and had a lower anxiety index (Figure 5F–H; *n* = 6 for shEphB2 + WAS or Sham‐virus + WAS group, one‐way ANOVA followed by Tukey's post hoc test or Kruskal–Wallis test; F, *F*_(2, 17)_ = 8.189, *p* = 0.0032; G, *F*_(2, 17)_ = 4.396, *p* = 0.0289; H, *p* = 0.0005). No difference was detected in time spent or entries made into the open arms, or anxiety index between rats in Sham‐WAS (control) and shEphB2 + WAS groups (Figure 5F–H; *n* = 8 for Sham‐WAS group, *n* = 6 for shEphB2 + WAS group). The results suggest that EphB2 knockdown in BLA also fails to induce anxiety‐like behaviors.

Compared with rats in Sham‐virus + WAS group, protein expression of EphB2 in BLA was successfully downregulated in shEphB2 + WAS group rats (Figure 5I,J; *n* = 4 for each group, unpaired Student's *t* test; J, *t*_(6)_ = 6.214, *p* = 0.0008). Furthermore, NR2A, NR2B, and PSD95 expressions also decreased in BLA in rats of shEphB2 + WAS group, but the expression of NR1 was unchanged between these two groups (Figure 5I,J; *n* = 4 for each group, unpaired Student's *t* test; J, NR2A, *t*_(6)_ = 8.008, *p* = 0.0002; NR2B, *t*_(6)_ = 8.829, *p* = 0.0001; PSD95, *t*_(6)_ = 5.038, *p* = 0.0024; NR1, Welch's correct, *t*_(3.043)_ = 0.3083, *p* = 0.7778).

Therefore, these data indicate that increase in EphB2 expression in BLA contributes to the induction of visceral hypersensitivity and anxiety‐like behaviors probably via an EphB2‐NMDARs‐PSD95 pathway in WAS rats.

### 3.6 EphB2 knockdown in BLA after WAS relieved visceral hypersensitivity and anxiety‐like behaviors

**FIGURE 6:**
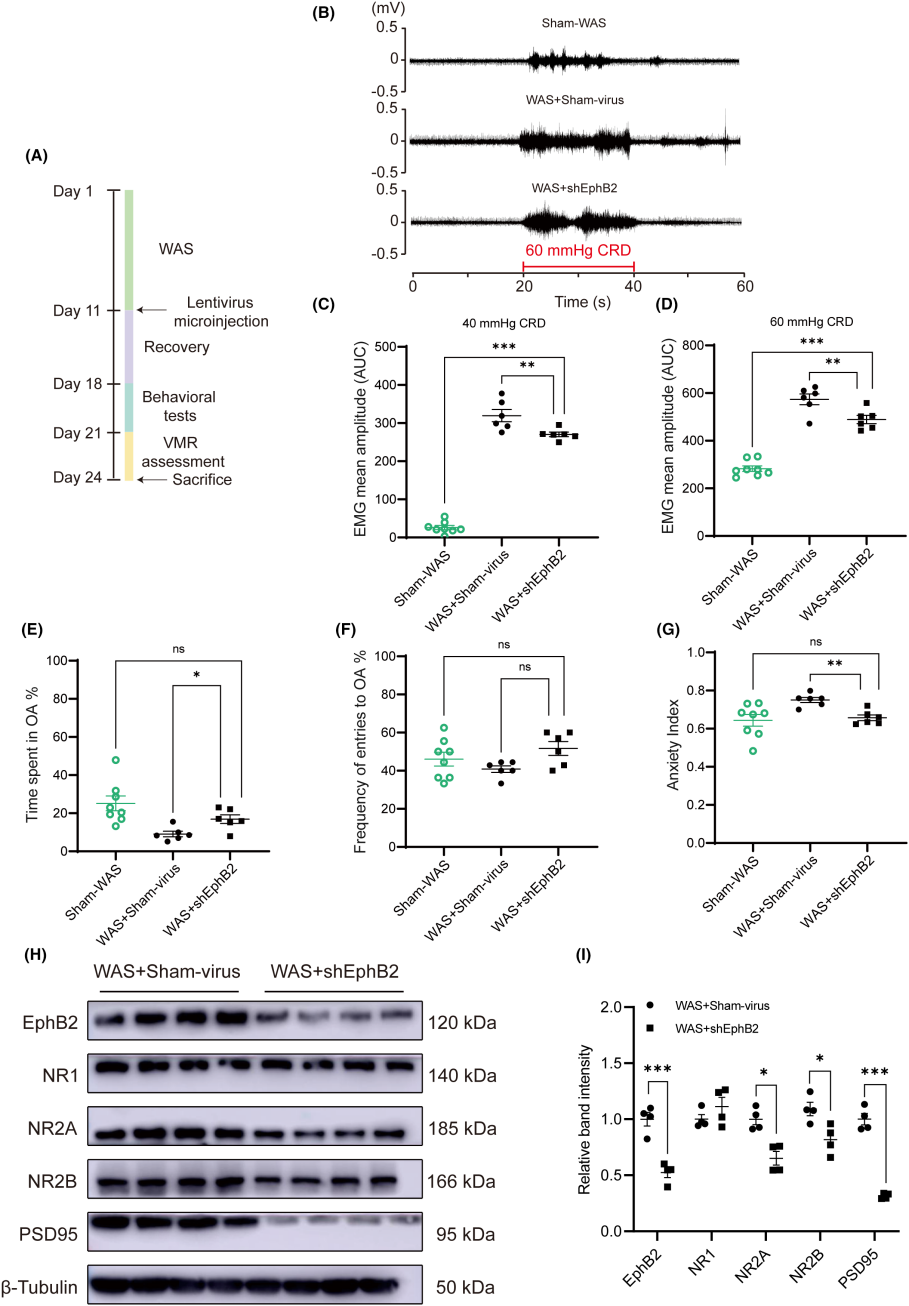
EphB2 knockdown in BLA after WAS partially alleviated visceral hypersensitivity but abolished anxiety‐like behaviors. (A) The schematic representation of the experimental protocol. (B) The representative EMG recordings of VMR amplitudes to 60 mmHg CRD. The VMR amplitudes to (C) 40 mmHg and (D) 60 mmHg CRD in rats of WAS + shEphB2 were lower than that of WAS + Sham‐virus group but were still higher than that of Sham‐WAS group. In EPM test, rats in WAS + shEphB2 group (E) spent more time in OA and (F) made similar entries into OA compared with that in WAS + Sham‐virus group. (G) Rats in WAS + shEphB2 group had a lower anxiety index than that in Sham‐virus + WAS group. There was no significant difference in anxiety‐like behaviors between rats in Sham‐WAS and WAS + shEphB2 groups. *n* = 8 for Sham‐WAS group, *n* = 6 for another groups. All data are given as mean ± SEM. *p < 0.05, **p < 0.01, ***p < 0.001, one‐way ANOVA followed by Tukey's or Dunnett's post hoc test. (H) Representative immunoblots showed the expression of EphB2, NR1, NR2A, NR2B, PSD95, and β‐Tubulin. (I) The protein expression of EphB2, NR2A, NR2B, and PSD95 in BLA was reduced in WAS + shEphB2 group compared with that in WAS + Sham‐virus group. *n* = 4 for each group. All data are given as mean ± SEM. *p < 0.05, **p < 0.01, ***p < 0.001, two‐tailed unpaired Student's *t*‐test or Mann–Whitney test.

Knocking down the EphB2 protein after WAS was performed to clarify the relationship between EphB2 in BLA and the maintenance of visceral hypersensitivity. The experimental protocol of this part is shown in Figure 6A. The virus spanned BLA from bregma −1.72 to −2.76 mm according to atlas.^35^ Compared with rats injected with Sham lentivirus in bilateral BLA after WAS (WAS + Sham‐virus group), rats microinjected with shEphB2 lentivirus after WAS (WAS + shEphB2 group) showed significantly lower visceral sensitivity too (Figure 6B–D; B, VMR amplitudes to 60 mmHg CRD; *n* = 6 for WAS + Sham‐virus or WAS + shEphB2 group, one‐way ANOVA followed by Tukey's post hoc test; C, *F*_(2, 17)_ = 288.6, *p* < 0.0001; D, *F*_(2, 17)_ = 84.82, *p* < 0.0001). However, rats in WAS + shEphB2 group still had higher VMR amplitudes to 40 or 60 mmHg CRD compared with that in Sham‐WAS group (control) (Figure 6C,D; *n* = 8 for Sham‐WAS group, *n* = 6 for WAS + shEphB2 group, one‐way ANOVA followed by Tukey's post hoc test; C, *F*_(2, 17)_ = 288.6, *p* < 0.0001; D, *F*_(2, 17)_ = 84.82, *p* < 0.0001). The results suggest that knocking down the protein of EphB2 in bilateral BLA after WAS only partially alleviates visceral hypersensitivity in WAS rats. EPM test revealed that rats in WAS + shEphB2 group had a reduction in anxiety‐like behaviors compared with that in WAS + Sham‐virus group, but there was no difference in anxiety‐like behaviors between Sham‐WAS (control) and WAS + shEphB2 groups (Figure 6E–G; *n* = 8 for Sham‐WAS group, *n* = 6 for WAS + Sham‐virus or WAS + shEphB2 group, one‐way ANOVA followed by Tukey's or Dunnett's post hoc test; E, Welch's correction, *p* = 0.0044; F, *F*_(2, 17)_ = 2.454, *p* = 0.1158; G, Welch's correction, *p* = 0.0017). The data of EPM suggest that EphB2 knockdown in BLA abolishes anxiety‐like behaviors in WAS rats.

Compared with rats in WAS + Sham‐virus group, EphB2 in BLA was successfully downregulated in WAS + shEphB2 group rats (Figure 6H,I; *n* = 4 for each group, unpaired Student's *t* test; I, EphB2, *t*_(6)_ = 6.361, *p* = 0.0007). Consistently, NR2A, NR2B, and PSD95 expressions also decreased in BLA in rats of WAS + shEphB2 group, but the expression of NR1 remained unchanged between the two groups (Figure 6H,I; *n* = 4 for each group, unpaired Student's *t* test or Mann–Whitney test; I, NR2A, *p* = 0.0286; NR2B, *t*_(6)_ = 3.107, *p* = 0.0209; PSD95, Welch's correct, *t*_(3.334)_ = 12.920, *p* = 0.0006; NR1, *t*_(6)_ = 1.234, *p* = 0.2634). The original blot images are provided in the supporting information (S1).

### 3.7 Activation of NMDARs following EphB2 knockdown induced visceral hypersensitivity and anxiety‐like behaviors

**FIGURE 7:**
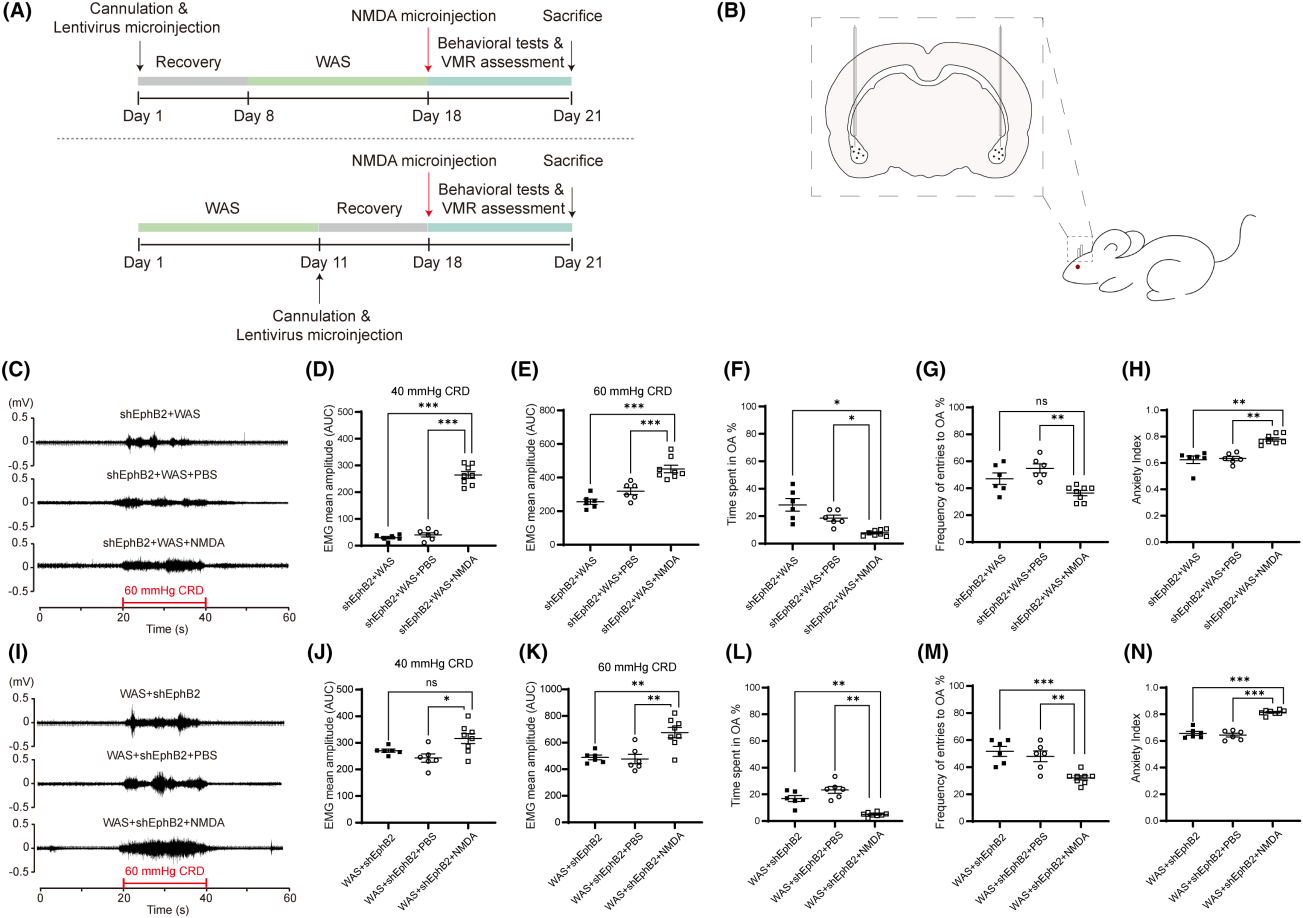
Activation of NMDARs following EphB2 knockdown resulted in visceral hypersensitivity and anxiety‐like behaviors. (A) The schematic representation of the experimental protocol. (B) The schematic drawing of cannulation targeting bilateral BLA. (C) The representative EMG recordings of VMR amplitudes to 60 mmHg CRD in shEphB2 + WAS, shEphB2 + WAS + PBS, and shEphB2 + WAS + NMDA groups. The VMR amplitudes to (D) 40 mmHg and (E) 60 mmHg CRD in rats of shEphB2 + WAS + NMDA were higher than that of shEphB2 + WAS and shEphB2 + WAS + PBS groups. EPM test showed that rats in shEphB2 + WAS + NMDA (F) spent less time, (G) had fewer entries into the open arms, and (H) had a higher anxiety index compared with that in shEphB2 + WAS + PBS group. *n* = 6 for shEphB2 + WAS or shEphB2 + WAS + PBS group, *n* = 8 for shEphB2 + WAS + NMDA group. All data are given as mean ± SEM. *p < 0.05, **p < 0.01, ***p < 0.001, one‐way ANOVA followed by Tukey's or Dunnett's post hoc test or Kruskal–Wallis test. (I) The representative EMG recordings of VMR amplitudes to 60 mmHg CRD in WAS + shEphB2, WAS + shEphB2 + PBS, and WAS + shEphB2 + NMDA groups. The VMR amplitudes to (J) 40 mmHg and (K) 60 mmHg CRD in rats of WAS + shEphB2 + NMDA were significantly higher than that of WAS + shEphB2 + PBS group. EPM test showed that rats in WAS + shEphB2 + NMDA (L) spent less time, (M) had fewer entries into the open arms, and (N) presented a higher anxiety index compared with that in the other two groups. *n* = 6 for WAS + shEphB2 or WAS + shEphB2 + PBS group, *n* = 8 for WAS + shEphB2 + NMDA group. All data are given as mean ± SEM. **p < 0.01, ***p < 0.001, one‐way ANOVA followed by Tukey's or Dunnett's post hoc test.

Agonist of NMDARs was used to confirm the role of NMDARs in WAS model after the knockdown of EphB2 expression. The experimental protocols are shown in Figure 7A. Schematic drawing of bilateral injections of lentivirus or NMDA into BLA is shown in Figure 7B. Compared with rats in shEphB2 + WAS and shEphB2 + WAS+PBS groups, rats with NMDA injection in BLA (shEphB2 + WAS + NMDA) showed significantly higher visceral sensitivity (Figure 7C–E; C, VMR amplitudes to 60 mmHg CRD; *n* = 6 for shEphB2 + WAS or shEphB2 + WAS + PBS group; *n* = 8 for shEphB2 + WAS+NMDA group, one‐way ANOVA followed by Tukey's or Dunnett's post hoc test; D, Welch's correction, *p* < 0.0001; E, *F*_(2, 17)_ = 24.65, *p* < 0.0001). In the EPM test, rats in shEphB2 + WAS+NMDA group spent less time and had fewer entries into the open arms, thus had a higher anxiety index compared with that in the other two groups (Figure 7F–H; *n* = 6 for shEphB2 + WAS or shEphB2 + WAS + PBS group, *n* = 8 for shEphB2 + WAS + NMDA group, one‐way ANOVA followed by Tukey's or Dunnett's post hoc test or Kruskal–Wallis test; F, Welch's correction, *p* = 0.0014; G, *F*_(2, 17)_ = 9.304, *p* = 0.0019; H, *p* < 0.0001).

On the other hand, after WAS and EphB2 downregulation procedures, rats with NMDA microinjection (WAS+shEphB2 + NMDA group) in BLA also showed higher visceral sensitivity than that in rats microinjected with PBS (WAS+shEphB2 + PBS group) or WAS+shEphB2 group (Figure 7I–K; I, VMR amplitudes to 60 mmHg CRD; *n* = 6 for WAS+shEphB2 or WAS+shEphB2 + PBS group, *n* = 8 for WAS+shEphB2 + NMDA group, one‐way ANOVA followed by Tukey's or Dunnett's post hoc test; J, Welch's correction, *p* = 0.0499; and K, *F*_(2, 17)_ = 11.43, *p* = 0.0007). The results of EPM test showed that rats in WAS+shEphB2 + NMDA group spent less time, had fewer entries into the open arms, and presented a higher anxiety index compared with that in the other two groups (Figure 7L–N; *n* = 6 for WAS+shEphB2 or WAS+shEphB2 + PBS group, *n* = 8 for WAS+shEphB2 + NMDA group, one‐way ANOVA followed by Tukey's or Dunnett's post hoc test; L, Welch's correction, *p* = 0.0002; M, *F*_(2, 17)_ = 13.21, *p* = 0.0003; N, *F*_(2, 17)_ = 74.31, *p* < 0.0001).

These data suggest that EphB2 knockdown inhibits visceral hypersensitivity via downregulation of NMDARs.

## 4 DISCUSSION

In IBS, chronic abdominal pain is a process associated with induction and maintenance of visceral hypersensitivity. Visceral hypersensitivity persists even though the initial pathogenic factors such as psychological stressors were removed.^33^ However, many studies only focused on the stage when chronic pain has been established but not its induction.^21^ In this study, we found the different roles of BLA and the associated molecular mechanism of EphB2 in the early and late stages of stress‐induced visceral hypersensitivity.

Imaging studies demonstrated that the activity of amygdala and its interconnected pain‐associated regions is higher in various chronic pain conditions, including in IBS.^36^, ^37^ Amygdala comprises a central nucleus (CeA), lateral nucleus, and BLA. Previous rodent studies in IBS mainly focused on CeA named as the “nociceptive amygdala,” which is critical in response to the hypothalamic–pituitary–adrenal axis.^20^, ^38^ Although BLA, a center for encoding negative emotions, was seldom investigated in visceral pain,^39^ emerging evidence has shown that it is also involved in modulation of pain.^18^, ^40^ For example, BLA inputs to the medial prefrontal cortex (mPFC) are critical in pain and related cognitive deficits.^41^, ^42^ The enhancement of long‐term depression at BLA‐CeA synapses is related to the pathogenesis of neuropathic pain and negative emotion.^22^ The present study demonstrated that neurons in BLA were significantly activated in rats with WAS‐induced visceral hypersensitivity, further suggesting that increased activity in BLA also participates in the modulation of visceral pain.^39^

Furthermore, by inactivating bilateral BLA before or after chronic WAS, we investigated the involvement of BLA in the induction and maintenance of visceral hypersensitivity in more detail. Previous studies have shown that poor socioeconomic state, neglect, and psychosocial trauma in early life are linked to developing IBS as well as severer symptoms, and resulted in poorer outcomes.^1^ Several studies showed that patients with IBS develop mood disturbance earlier than the onset of bowel symptoms (diarrhea or constipation), implying the important role of mood disturbance in IBS development.^43^ It has been revealed that psychological distress influences visceral pain in IBS by central pain sensitization and the information is carried through vagal and sympathetic efferent in a top‐down model.^7^, ^44^ In this study, we found that BLA inactivation prior to WAS failed to induce visceral hypersensitivity in WAS rats, which is consistent with the idea that stress‐inducing visceral pain sensitization depends on top‐down modulation.^7^, ^43^, ^44^ On the other hand, after visceral sensitization was established, our results showed that visceral hypersensitivity was still partially inhibited by inactivation of BLA, implying the involvement of interaction between gut and BLA in maintaining visceral pain sensitization. Furthermore, some other brain regions such as CeA, mPFC, or insular cortex may also participate in maintaining visceral hypersensitivity,^14^ which merits further investigation. Besides, it is not surprising that inactivation of BLA abolished anxiety‐like behaviors due to its role as an integrative center in regulating anxiety responses.^40^

The Eph RTKs family consists of 14 identified transmembrane proteins which are divided into EphA and EphB receptor subfamilies. Eph receptors localize mainly at both pre‐ and postsynapses and participate in not only brain development but also the regulation of synaptic functions.^24^ Moreover, increasing evidence has revealed the importance of Eph receptors in chronic pain modulation.^29^, ^31^, ^45^ Among these receptors, it has been found that overexpression of EphB2 is critical in both psychiatric disorders^27^, ^46^ and neuropathic pain.^29^ In the studies of IBS, EphB2 in enteric nerves was recently found to be critical in sustained visceral hypersensitivity in both patients and rats.^30^, ^31^ An analysis of seven Eph receptors expressed abundantly in BLA was carried out to investigate the role of Eph receptors in visceral hypersensitivity. Consistently, we found that only EphB2 was upregulated no matter in mRNA or protein levels in WAS rats. Furthermore, we found knocking down EphB2 in BLA before WAS could abolish the induction of visceral hypersensitivity and anxiety‐like behaviors. Interestingly, knocking down EphB2 in BLA after WAS model establishment could abolish anxiety‐like behaviors but only partially relieve visceral hypersensitivity during the maintenance stage of chronic visceral pain. The different effects of EphB2 expression in BLA were consistent with the results of BLA lesion in different phases of visceral hypersensitivity, strongly indicating that EphB2 in BLA acts as a significant molecule in process of stress‐induced visceral hypersensitivity, especially at the early induction stage.

Growing evidence has shown that synaptic plasticity such as LTP is crucial to the development and maintenance of visceral hypersensitivity.^47^, ^48^ NMDARs are ionotropic glutamate receptors whose activation is essential for synaptic plasticity.^49^ Additionally, many studies have found that EphB2 and NMDARs are co‐localized and interact with each other.^50^, ^51^ In the mammalian DRG neurons, EphB2 plays its function through either directly facilitating calcium influx or in a NMDARs‐dependent manner.^28^, ^52^ Moreover, the results in the present study showed that in WAS rats with EphB2 knocking down, NMDA microinjection into bilateral BLA successfully induced visceral hypersensitivity and anxiety‐like behaviors, indicating that EphB2 knockdown inhibits visceral hypersensitivity via downregulation of NMDARs. As predominant components of functional NMDA‐type receptor channels, NR1 and NR2A/NR2B subunits are involved in visceral hypersensitivity.^53^ EphB2 increases tyrosine phosphorylation of NR2B through its Src tyrosine kinase activity and then regulates synaptic plasticity, resulting in hyperalgesia.^54^, ^55^ A study based on metabolomics and proteomics also showed that upregulated EphB2 might facilitate the cluster of NR2A.^56^ On the other hand, there is evidence that suggests that EphB2 directly binds to the extracellular region of NR1 subunit, which helps to anchor NMDARs at postsynaptic membrane in neurons.^50^, ^51^ However, the change in NR1 expression was inconsistent in different studies of visceral hypersensitivity. It has been reported that NR1 increases in visceral hypersensitivity induced by colitis^57^ but it remains unchanged in some models induced by emotional stress such as maternal separation^33^ or restraint stress.^46^ Further study has shown that restraint stress leads to dissociation of EphB2 from NR1 subunit in amygdala, thus enhancing NMDA current,^46^ which suggests the link of EphB2‐NR1 interaction with NMDA current. In this study, we found the protein level of NR2A and NR2B but not NR1 increased in BLA in WAS rats. After EphB2 knockdown in WAS rats, expression of both NR2A and NR2B was downregulated. However, the knocking down of EphB2 in BLA failed altering the expression of NR1. These results support that EphB2 regulates WAS‐associated visceral hypersensitivity by increasing NR2, but not NR1 expression. However, the involvement of EphB2‐NR1 interaction could not be excluded. PSD95, a critical postsynaptic protein for postsynaptic plasticity is strongly correlated with visceral hypersensitivity.^58^ The expression of PSD95 was increased following the overexpression of EphB2. Knocking down of EphB2 also downregulated the expression of PSD95 in WAS rats, suggesting that EphB2 exerted a great influence on synaptic plasticity.

To avoid the possible effects of sex hormones on pain perception and emotion, experiments were performed only on male rats in the present study.^59^, ^60^ However, the single‐sex experimental design perhaps limits the observation of female subjects. Since IBS is predominant in females and menstrual cycles are associated with symptom severity,^61^, ^62^ the sex difference in EphB2‐NMDA pathway in WAS‐induced visceral pain sensitization is significant and deserves further exploration. In addition to CRD‐evoked VMR recording used in this study to assess visceral sensation of the colon/rectum, sensitized colon/rectum motility including contractile activity^63^ or fecal pellet output numbers^64^ is also used as an indicator of visceral hypersensitivity. Therefore, the changes in colon/rectum motility are worth testing in further studies. Moreover, besides using CRD to reflect colon/rectal hypersensitivity of IBS, some other methods such as von Frey filaments test can be used to assess visceral pain (bladder or uterus pain).^65^, ^66^

Nevertheless, despite the above limitations, this study demonstrated that chronic psychological stress increased EphB2 expression in BLA and may enhance synaptic plasticity through upregulating NMDARs, which results in visceral hypersensitivity and anxiety‐like behaviors. These results provide evidence that EphB2 in BLA is essential for visceral hypersensitivity induction by anxiety stress, which implies that early treatment for patients with psychiatric disorders is important to prevent the development of IBS.

## FUNDING INFORMATION

The work was supported by the National Natural Science Foundation of China (grant nos. 81974067 and 31972914) and the Shanghai Science and Technology Innovation Action Plan (grant nos. 22Y11908300 and 22140902200).

## CONFLICT OF INTEREST STATEMENT

The authors declare no conflicts of interest.

## Supporting information


Data S1

## Data Availability

The data used and/or analyzed during the present study are available from the corresponding authors upon reasonable request.
