# Supplementary material for: Overexpression of EphB2 in the basolateral amygdala is crucial for inducing visceral pain sensitization in rats subjected to water avoidance stress
Source: CNS Neurosci Ther. 2024 Feb 14;30(2):e14611. doi: 10.1111/cns.14611 (PMC10865153; doi:10.1111/cns.14611)

Full unedited gel/blot for Figure 4D

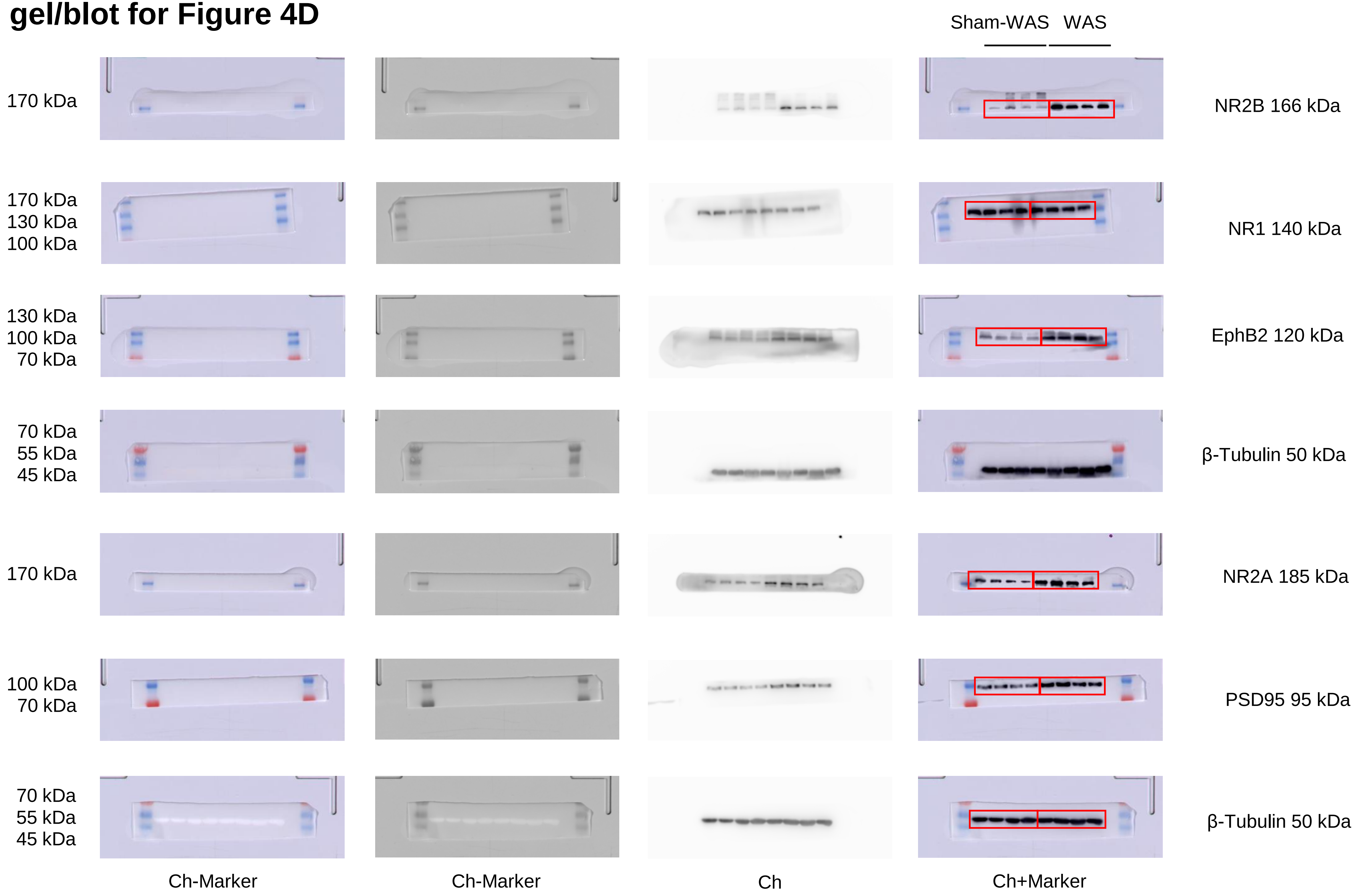

Full unedited gel/blot for Figure 5I

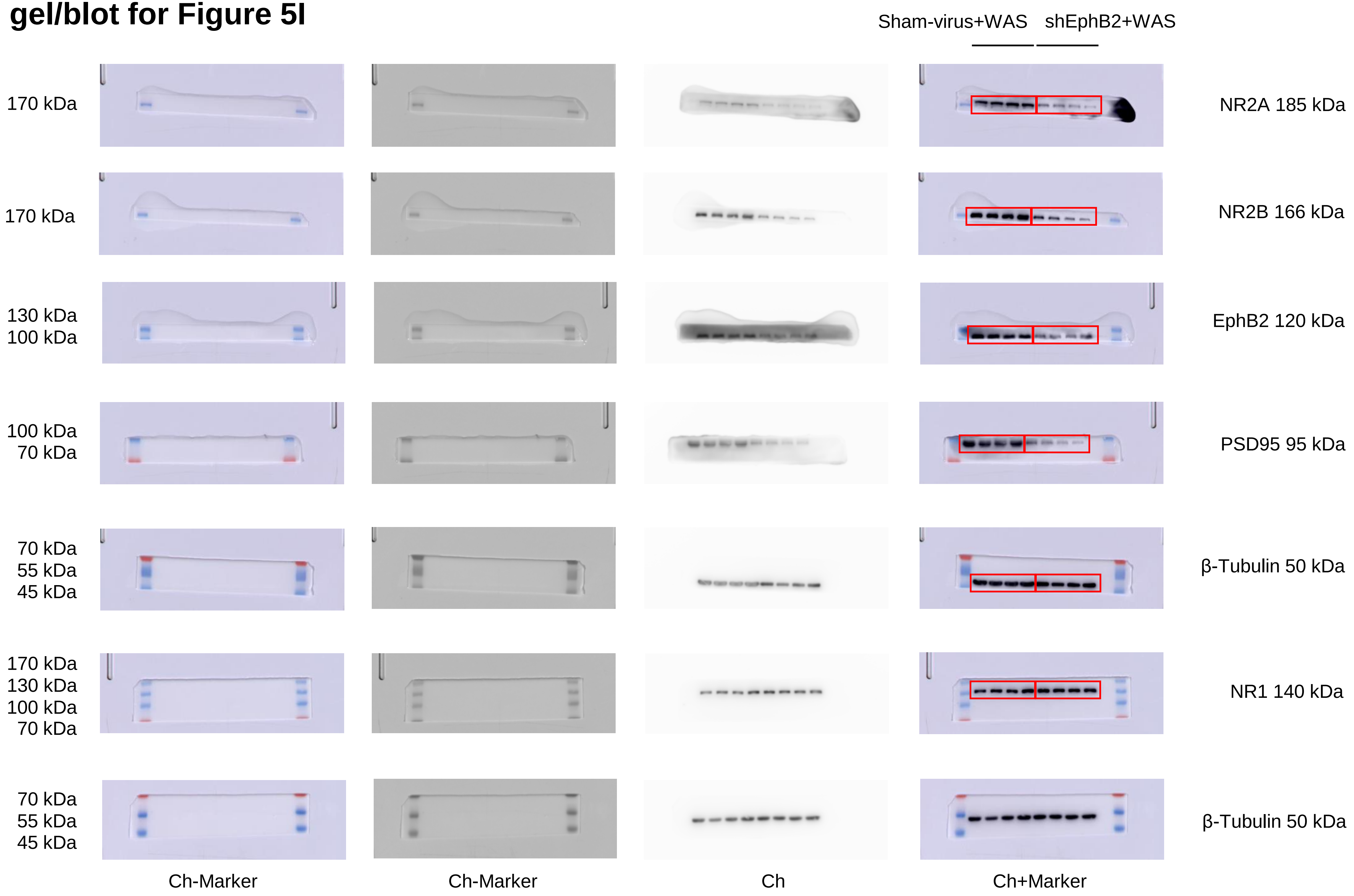

Full unedited gel/blot for Figure 6H

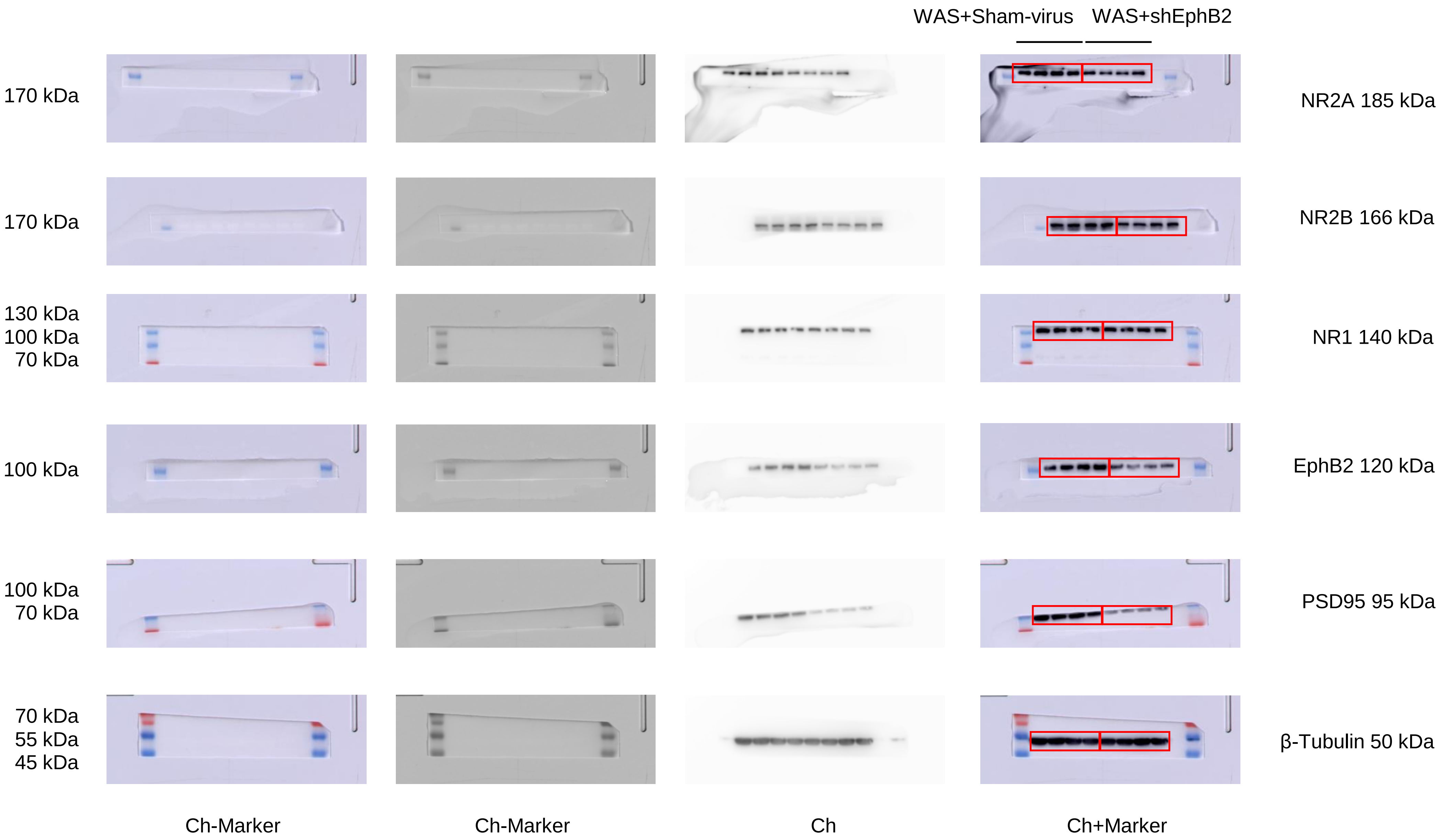

Supplement: Supplementary file 1 — Data S1 [file CNS-30-e14611-s001.pdf]
